# Dermoscopy of Anserine Folliculosis

**DOI:** 10.5826/dpc.1102a19

**Published:** 2021-03-08

**Authors:** Shekhar Neema, Shivam Goyal

**Affiliations:** 1 Department of Dermatology, Armed Forces Medical College, Pune, India; 2 Department of Dermatology, Kasturba Medical College, MAHE, Manipal, India

**Keywords:** lichen nitidus, anserine folliculosis, keratosis pilaris, dermoscopy

## Introduction

Anserine folliculosis is a rare dermatological entity presenting with multiple closely grouped follicular-oriented papules on the chin, neck, and jaw. It is difficult to clinically differentiate it from other folliculocentric keratotic disorders like keratosis pilaris, lichen nitidus, follicular mucinosis, and folliculotropic mycosis fungoides (FMF). Dermoscopy can provide an essential clue in the diagnosis of anserine folliculosis and differentiate it from other known entities.

## Case Presentation

**Figure 1:**
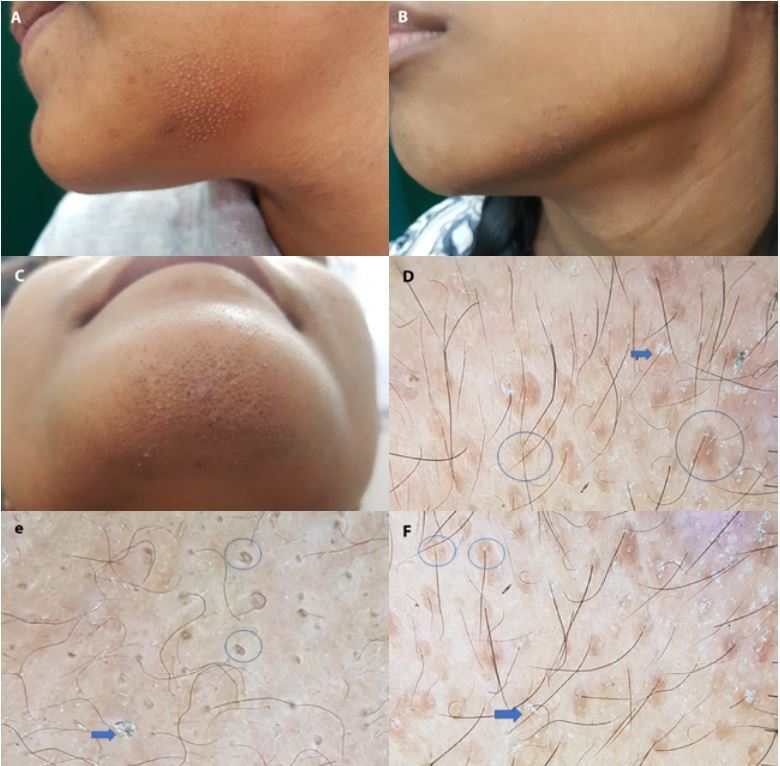
Clinical images show: (A) grouped follicular, skin-colored papules on the left jawline; (B) grouped follicular papules on left jawline; and (C) grouped follicular papules on chin and jawline. Dermoscopy of anserine folliculosis shows: (D) white scales (blue arrow), patulous follicles and yellow dots (blue circle) (DermLite DL4, polarized, ×10); (E) white scales (blue arrow), patulous follicles and yellow dots (blue circle) (DermLite DL4, polarized, ×10) and (F) white scales (blue arrow), patulous follicles and yellow dots (blue circle) (DermLite DL4, polarized, ×10).

**Figure 2:**
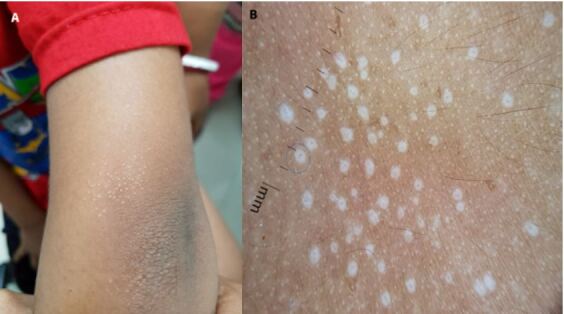
(A) Clinical image shows grouped shiny white papules on the left elbow. (B) Dermoscopy of lichen nitidus shows a white structureless area with a central brown area (blue circle). These structures interrupt underlying skin markings (DermLite DL4, polarized, ×10).

**Figure 3:**
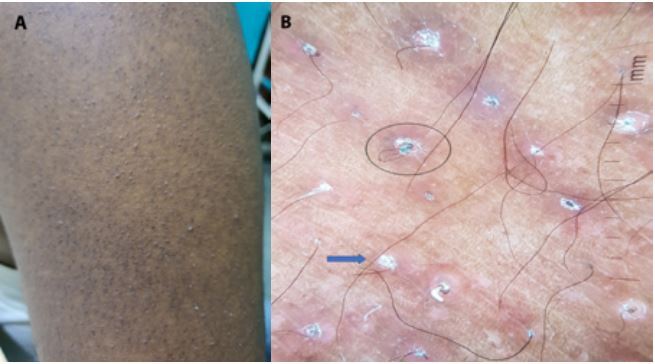
(A) Clinical image shows skin-colored to hyperpigmented follicular papules with background hyperpigmentation over the left arm. (B) Dermoscopy of keratosis pilaris shows twisted hair and peripilar cast (blue circle). Erythema is present surrounding the follicle (blue arrow) (DermLite DL4, polarized, ×10)

Here, we present 3 cases with rough lesions on the chin or jaw of varying duration (Table 1). History of repeated friction in the area while studying was present. Cutaneous examination revealed multiple, closely-set, grouped, skin-colored, follicular-oriented papules on the chin, which felt like sandpaper on palpation (Figure 1, A–C). General and systemic examination revealed no abnormality. A diagnosis of anserine folliculosis was made based on presentation. Dermoscopy revealed white scales, yellow dots, and patulous follicular openings (Figure 1, D–F). Lichen nitidus, keratosis pilaris, follicular mucinosis, and FMF are other differential diagnoses of this presentation. Dermoscopy was compared with features of other conditions (Figures 2B and 3B). Histopathological confirmation could not be done because the patients refused. All 3 patients were treated with topical tretinoin 0.025% cream and advised to restrict friction.

Anserine folliculosis mostly presents in a younger age group with follicular papules on the chin, neck, or jaw and has a sandpaper or goose skin-like feeling on touch. The patients usually give a history of friction or trauma, which has been considered a possible etiologic factor. However, it is not found in all cases. The presence of atopy has been linked to the incidence of anserine folliculosis. Dermoscopic signs have not been elucidated to date. Histopathological features include epidermis with hyperkeratosis, hypergranulosis, the focal presence or increase of the stratum lucidum, rudimentary follicles, and dilatation of follicular openings with retention of keratotic material [1]. Hyperkeratosis corresponds to white scales on dermoscopy and dilatation of follicular openings with retention of keratotic material corresponding to patulous follicles and yellow dots. This condition can be compared to its close differentials, lichen nitidus, and keratosis pilaris. The absence of histopathological analysis is a limitation in our case series.

Lichen nitidus presents as multiple, tiny, discrete, shiny papules in clusters over the flexor aspect of upper extremities, genitalia, and anterior trunk (Figure 2A). Dermoscopy shows multiple white, well-circumscribed, circular areas with a smooth surface, and a brownish shadow inside the circle [2] (Figure 2B).

Keratosis pilaris presents as keratotic follicular plugs with or without perifollicular erythema over the extensor aspect of forearms, thighs, trunk, buttocks, and face (Figure 3A). Dermoscopic features are twisted or coiled vellus surrounded by peripilar cast or scaling with or without perifollicular erythema and pigmentation (Figure 3B).

Primary follicular mucinosis presents as one or several pink plaques, often composed of grouped follicular papules. Dermoscopy features are dilated follicular ostia with follicular plugs of amorphous material. FMF presents as follicle-based patches, plaques, infiltrated plaques, tumors, and prurigo nodularis-like lesions, as well as keratosis pilaris-like lesions and acneiform lesions mostly on the head and neck region. Dermoscopy shows dilation of follicular openings with partial destruction of hair follicles, orange-pink perifollicular clods, yellowish background, white/pigmented halos, white clods/structureless areas or short fine vessels, or a combination of these. These features were not seen in our cases.

## Conclusion

Keratotic papules on the chin are a diagnostic challenge. Anserine folliculosis is a rare entity that presents with similarities to follicular-oriented conditions. Dermoscopy can aid in the diagnosis of this condition.

## Figures and Tables

**Table 1:** Case Demographics and Clinical Features

Cases	Age	Sex	Clinical Feature	Duration
Case 1	18	Female	Grouped follicular papule, left jawline	2 months
Case 2	16	Female	Grouped follicular papule, left jawline	2½ months
Case 3	21	Female	Grouped follicular papules on chin	3 months
